# Identifying possible biomarkers of lower urinary tract symptoms using metabolomics and partial least square regression

**DOI:** 10.1007/s11306-023-02046-2

**Published:** 2023-09-12

**Authors:** Florin V Hopland-Nechita, John R Andersen, Tarja Kvalheim Rajalahti, Trygve Andreassen, Christian Beisland

**Affiliations:** 1 Department of Urology, Førde Central Hospital, Førde, Norway; 2 Department of Clinical Medicine, University of Bergen, Bergen, Norway; 3 Western Norway University of Applied Sciences, Førde, Norway; 4 Department of Chemistry, University of Bergen, Bergen, Norway; 5 Department of Circulation and Medical Imaging, Norwegian University of Science and Technology, Trondheim, Norway; 6 Central staff, St. Olavs Hospital HF, Trondheim, Norway; 7 Department of Urology, Haukeland University Hospital, Bergen, Norway

**Keywords:** LUTS, Metabolomics, BPH, Biomarker, PLS

## Abstract

**Introduction:**

The *objective* of this study was to explore potential novel biomarkers for moderate to severe lower urinary tract symptoms (LUTS) using a metabolomics-based approach, and statistical methods with significant different features than previous reported.

**Materials and Methods:**

The patients and the controls were selected to participate in the study according to inclusion/exclusion criteria (n = 82). We recorded the following variables: International prostatic symptom score (IPSS), prostate volume, comorbidities, PSA, height, weight, triglycerides, glycemia, HDL cholesterol, and blood pressure. The study of 41 plasma metabolites was done using the nuclear magnetic resonance spectroscopy technique. First, the correlations between the metabolites and the IPSS were done using Pearson. Second, significant biomarkers of LUTS from metabolites were further analysed using a multiple linear regression model. Finally, we validated the findings using partial least square regression (PLS).

**Results:**

Small to moderate correlations were found between IPSS and methionine (-0.301), threonine (-0.320), lactic acid (0.294), pyruvic acid (0.207) and 2-aminobutyric-acid (0.229). The multiple linear regression model revealed that only threonine (p = 0.022) was significantly associated with IPSS, whereas methionine (p = 0.103), lactic acid (p = 0.093), pyruvic acid (p = 0.847) and 2-aminobutyric-acid (p = 0.244) lost their significance. However, all metabolites lost their significance in the PLS model.

**Conclusion:**

When using the robust PLS-regression method, none of the metabolites in our analysis had a significant association with lower urinary tract symptoms. This highlights the importance of using appropriate statistical methods when exploring new biomarkers in urology.

## Introduction

Benign prostatic hyperplasia (BPH) is a prominent urological issue among elderly men in various nations, serving as a major etiology for lower urinary tract symptoms (LUTS) in this population. Descriptive epidemiological studies have reported varying prevalence rates of BPH, ranging from 12–42%(Lee, Chan, & Lai, 2017), with one study estimating a lifetime risk of 36.6%(Wang, Guo, Zhang, Tian, & Zhang, 2015). Globally, the number of prevalent BPH cases was estimated at 940 million in 2019, compared to 511 million in 2000. The substantial increase of 70.5% in the global prevalence of BPH can predominantly be ascribed to population ageing. This emerging trend, coupled with the escalating life expectancy worldwide, is projected to further augment the overall burden of BPH. The disease has been linked to increased healthcare costs and diminished quality of life (GBD, 2022). It is associated with various serious complications, including a higher risk of falls, depression, and reduced health-related quality of life (HRQoL) indicators such as sleep, psychological well-being, daily activities, and sexual health. The impact of BPH extends beyond the patient to their family and society.(Speakman, Kirby, Doyle, & Ioannou, 2015) Consequently, there is a pressing need for proactive monitoring and planning to address the potential strain on healthcare systems. Additionally, this situation underscores the necessity for improved patient stratification, considering the most effective treatment strategies, and adopting personalized medicine approaches (Feinstein, 2018).

Biomarkers for BPH can potentially distinguish between different BPH-related conditions. Currently, these conditions are primarily identified based on symptoms. Biomarker research aims to identify the risk of disease progression and guide the development of earlier, personalized, efficient, and cost-effective medical approaches for managing BPH-related LUTS. Furthermore, these efforts in biomarker discovery could provide a new understanding of the molecular causes of histological BPH and the clinical presentations of BPH, which have remained unclear despite ongoing research(Mullins et al., 2008).

The serum prostate-specific antigen (PSA) is the only available biomarker in clinical practice (Cornu J.N., 2023). Pooled analyses of randomized controlled trials (RCTs) involving men with LUTS and presumed benign prostatic obstruction (BPO) have demonstrated that PSA exhibits good predictive value for assessing prostate volume, prostate growth, changes in symptoms, quality of life (QoL)/bother and clinical progression to urinary retention (Kozminski, Wei, Nelson, & Kent, 2015; Patel et al., 2018; Roehrborn, 2008).

In a recent meta-analysis, the collective evidence regarding microRNAs (miRNAs) involved in the pathogenesis of BPH was reviewed. The analysis highlighted miR-221 as a notably associated miRNA with BPH, indicating its potential as a biomarker and therapeutic target for early detection and management of the condition.(Greco et al., 2019).

The term “metabolome” was initially introduced by Oliver et al. in 1998 to describe the complete collection of low molecular weight compounds found within a cell, which are necessary for its maintenance, growth, and normal function. The metabolome also contributes to the metabolic reactions occurring within a cell during specific physiological or developmental stages(Oliver, Winson, Kell, & Baganz, 1998). Metabolomics, an analytical chemistry method, aims to measure a portion or the entirety of the metabolome. It can potentially discover new biomarkers that can predict the incidence, severity, and progression of diseases and identify underlying pathophysiological abnormalities (Newgard, 2017). Initially, metabolomics primarily focused on biomarker discovery (Johnson, Ivanisevic, & Siuzdak, 2016). Consequently, plasma trimethylamine N-oxide (TMAO) and urinary taurine have been identified as markers for cardiovascular disease (Koeth et al., 2013). A link has been established between dysregulated metabolism of branched-chain amino acids (BCAAs) and various cardiometabolic diseases (Newgard, 2017). In 2018, Mitsui et al. conducted the solitary metabolomics study focused on male LUTS. They postulated that the abnormal glucose metabolism observed in patients with metabolic syndrome might similarly manifest in those with LUTS. Such a finding, if validated, could shed light on modifications in the amino acid profiles of plasma, thereby unveiling potential pathways for the development of innovative LUTS treatments (Mitsui et al., 2018). However, the statistical methodologies employed in that study were questioned for their robustness and reliability.

The primary aim of our research was twofold: First, to validate and replicate the findings of Mitsui et al.‘s pioneering metabolomics study on male LUTS secondary to BPH. And second, to bolster the validity of any findings by employing the partial least squares (PLS) regression, a statistical method widely acknowledged as the gold standard in metabolomics research. Through this approach, we seek to reinforce the findings from the 2018 study, potentially opening doors to new research directions and treatment approaches.

## Material and method

A cross-sectional study was conducted at the Urologic Outpatient Clinic of Førde Central Hospital, with an inclusion period spanning from November 20, 2018, to February 17, 2021. Patients were referred by their general practitioners (GPs). They were selected by the first author based on strict inclusion and exclusion criteria. Our research group previously published the study protocol (Hopland-Nechita, Andersen, & Beisland, 2022). At the time of inclusion, none of the enrolled patients were undergoing medical treatment for LUTS. The control group consisted of patients referred to the Surgical Outpatient Clinic of Førde Central Hospital for conditions other than LUTS, who consented to participate in the study. Detailed medical histories and lists of current medications were obtained, and the comorbidities were quantified using the Charlson index and ASA (American Society of Anesthesiology) scores.

*Blood samples* were collected from fasting patients in the morning, between 08:00 and 09:00 a.m. The immediate analysis included regular blood tests such as PSA, cholesterol, triglycerides, and creatinine. Serum was obtained following a standardized protocol: (i) Blood plasma was collected in 5 ml tubes containing gel (Vakuette® Serum Gel with activator). (ii) The tubes were gently inverted five times and placed vertically for coagulation. (iii) After 30 min, the sample was centrifuged at 2000xg for 10 min. Visual inspection of the serum was conducted for any residues, and if present, the centrifugation process was repeated. (iv) The serum tube was refrigerated at 4 °C until 0.5 ml aliquots were pipetted into cryotubes. (v) Finally, the cryotubes were stored at -80 °C.

### Method for metabolomics analysis

Serum metabolites were analyzed using proton NMR (nuclear magnetic resonance) spectroscopy (600 MHz instrument, Bruker Biospin) according to the procedure described in the literature (Dona et al., 2014). A total of 41 serum metabolite concentrations were automatically quantified using the commercial Bruker B.I. Quant-PS2.0™ methods from Bruker Boispin, based on algorithms developed for fitting predefined proton signals (Jiménez et al., 2018). Advanced data analytical tools using latent variable methods, described in detail in Rajalahti et al.(Rajalahti et al., 2009, 2010), are employed for analyzing the resulting spectra. These tools enable the detection of biomarker signatures from complex spectral profiles with higher reliability and make interpreting the results more accessible.

### Statistical analysis

In alignment with the procedures outlined in the prior study by Mitsui et al., the analysis of patient characteristics was conducted using the Student t-test. Pearson correlation was utilized to determine the relationship between the metabolites and the International Prostate Symptom Score (IPSS). Following this, a t-test comparison was executed between the LUTS and control groups without any adjustments. For metabolites that demonstrated statistically significant differences (p < 0.05) between the LUTS and control groups, a multiple linear regression analysis was then conducted, adjusting for age as well as comorbidities as measured by the Charlson index and the ASA score. All results were reported with two-tailed P-values and accompanied by 95% confidence intervals .

While the previous study by Mitsui et al. did not employ specific biomarker statistical analysis techniques, our research aimed to validate and further investigate the relationship between the IPSS and the explanatory variables. To achieve this, we utilized partial least squares (PLS) regression, following the methodology outlined by Rajalahti and Kvalheim(Rajalahti & Kvalheim, 2011). The PLS regression method decomposes the explanatory variables into PLS components, which are linear combinations of the original variables maximizing the covariance with the outcome variable. This technique is particularly suitable for handling multicollinear variables and adjusting for multiple comparisons. To validate the models, Monte Carlo resampling was performed with 100 repetitions, randomly selecting 50% of the observations as an external validation set for each repetition. Target projection (TP) was applied to the obtained PLS models to calculate selectivity ratios (SR) for each explanatory variable, following the methodology outlined by Rajalahti et al. (Rajalahti et al., 2009)

The statistical software IBM SPSS Statistics Version 26 was used for the standard statistical analyses, while Sirius 11.5 (Pattern Recognition Systems AS) was utilized for PLS analysis.

The project received approval from the Norwegian South-East Regional Ethics Committee (REC reference number: 2018/114). In accordance with the approved protocol, all participating patients provided informed consent before their inclusion in the study.

## Results

Out of the 169 patients who met the inclusion criteria, 91 (53%) provided informed consent to participate in the study by signing the required forms. Eight patients were subsequently excluded from the analysis, including five with a diagnosis of prostate cancer, one with a diagnosis of acute leukaemia, one with a diagnosis of bladder cancer, and one who withdrew their informed consent within one month of the inclusion date. Consequently, a total of 83 patients were included in the statistical analysis. The general characteristics of these patients are presented in Table 1.

**Table 1:** Patient characteristics

Variables	Control Group (IPSS < 8) n = 20	LUTS Group (IPSS ≥ 8) n = 63	P-value
Age (years)	63.4 (7.1)	64.4 (6.3)	0.546
IPSS, mean (SD)	4.3 (2.5)	18.3 (5.5)	< 0.001
ASA Classification, n (%)	1: 14 (70%) 2: 5 (25%) 3: 1 (5%)	1: 21 (33.3%) 2: 39 (61.9%) 3: 3 (4.8%)	0.013
Charlson Index, n (%)	0: 13 (65%) 1: 6 (30%) 2: 1 (5%)	0: 43 (68.3%) 1: 13 (20.6%) 2: 7 (11.1%)	0.807
Prostate Volume (cm^3^), mean (SD)	56.7 (28.4)	52.1 (22.6)	0.466
Residual Urine (ml), mean (SD)	81.6 (36.2)	101.7 (131.8)	0.573
Q-max (ml/sec), mean (SD)	17.7 (11.4)	15.8 (8.8)	0.475
PSA (µg/L). mean (SD)	3.5 (3.4)	3.1 (3)	0.608
BMI (kg/m^2^), mean (SD)	26.9 (3.2)	27.3 (4)	0.710

Note: IPSS: International Prostatic Symptom Score, LUTS: Lower urinary tract symptoms, ASA: American Society of Anestesiology, PSA: Prostatic Specific Antigen, BMI: Body Mass Index.

The patient cohort included in this study is representative of individuals referred to urologists for LUTS examinations. Conversely, the control group serves as a typical representation of men aged 50 to 80 years in Norway.

Following NMR analysis, 41 metabolites were identified and considered for further statistical analysis. The analysis revealed significant Pearson correlations between the IPSS and several metabolites (Table 2). Specifically, there was a negative correlation between IPSS and methionine (r = -0.301, p = 0.006) and threonine (r = -0.32, p = 0.003). On the other hand, a positive correlation was observed between IPSS and lactic acid (r = 0.294, p = 0.007) and 2-aminobutyric acid (r = 0.229, p = 0.038). However, the correlation between IPSS and pyruvic acid was not statistically significant (r = 0.207, p = 0.062).

**Table 2:** Pearson correlations between metabolites and the IPSS

IPSS	Methionine [mmol/L]	Threonine [mmol/L]	Lactic acid [mmol/L]	Pyruvic acid [mmol/L]	2-Aminobutyric acid [mmol/L]
Pearson correlation	-0.301	-0.32	0.294	0.207	0.229
P-value	0.006	0.003	0.007	0.062	0.038

Note: IPSS: International Prostatic Symptom Score

The results of the t-test (Table 3) indicate significant differences between the Control Group and LUTS Group for the variables methionine (p = 0.002), threonine (p = 0.038), lactic acid (p = 0.031), and 2-aminobutyric acid (p = 0.043). However, no statistically significant difference was observed for pyruvic acid (p = 0.194).

**Table 3:** Metabolites in LUTS versus control group

Variables, mean (SD)	Control Group (IPSS < 8) n = 20	LUTS Group (IPSS ≥ 8) n = 62	P-value
Methionine [mmol/L]	0.04 (0.02)	0.02 (0.02)	0.002
Threonine [mmol/L]	0.15 (0.1)	0.09 (0.09)	0.038
Lactic acid [mmol/L]	1.63 (0.36)	1.87 (0.44)	0.031
Pyruvic acid [mmol/L]	0.05 (0.02)	0.05 (0.03)	0.194
2-Aminobutyric acid [mmol/L]	0.01 (0.02)	0.03 (0.02)	0.043

Note: IPSS: International Prostatic Symptom Score; LUTS: Lower urinary tract symptoms

Only threonine (p = 0.022) demonstrated a significant association with the IPSS in the multiple linear regression model. However, the variables methionine (p = 0.103), lactic acid (p = 0.093), pyruvic acid (p = 0.847), and 2-aminobutyric acid (p = 0.244) did not retain their statistical significance with the IPSS (Table 4).

**Table 4:** Multiple linear regression model with the IPSS as the dependent variable

Variables	B (95% CI)	Beta	p-value
Age	-0.01 (-0.3–0.26)	-0.01	0.898
Charlson index	-1.91 (-4.65–0.83)	-0.17	0.168
ASA	3.14 (-0.43–7.72)	0.23	0.084
Methionine [mmol/L]	-74.31 (-163.94–15.32)	-0.18	0.103
Threonine [mmol/L]	-20.91 (-38.66 – -3.17)	-0.26	0.022
Lactic acid [mmol/L]	4.55 (-0.77–9.88)	0.25	0.093
Pyruvic acid [mmol/L]	-7.87 (-88.76–73)	-0.02	0.847
2-Aminobutyric acid [mmol/L]	46.74 (-32.51–125.99)	0.12	0.244

Note: Adjusted R^2^ = 0.192. IPSS: International Prostatic Symptom Score.

The cross-validated PLS regression analysis revealed no valid PLS components associated with IPSS. Therefore, we could not report reliable explained variance or selectivity ratios with 95% CIs for the individual explanatory variables.

## Discussion

This study aimed to investigate potential novel biomarkers for moderate to severe LUTS utilizing a metabolomics-based approach and employing statistical methods with distinct features from previous studies. The findings revealed moderate correlations between the IPSS and several metabolites, including methionine, threonine, lactic acid, pyruvic acid, and 2-aminobutyric acid. However, in the multiple linear regression model, only threonine exhibited a significant association with IPSS. Interestingly, none of the analyzed metabolites demonstrated a significant association with LUTS when utilising the robust PLS-regression method.

The small to moderate correlations between IPSS and some metabolites, including methionine, threonine, lactic acid, pyruvic acid, and 2-aminobutyric acid, suggest that these metabolites may play a role in the pathophysiology of LUTS. However, the lack of significance in the PLS model highlights the need for more extensive studies using larger sample sizes and a broader range of metabolites to confirm or refute these findings. Additionally, the lack of significance in the PLS model emphasizes the importance of using appropriate statistical methods when exploring new biomarkers in urology.

The significant association between lower threonine serum levels and IPSS in the multiple linear regression model suggests that threonine may be a potential biomarker for LUTS. Threonine is an essential amino acid involved in protein synthesis, immune function, energy metabolism and collagen production(Tang, Tan, Ma, & Ma, 2021). It is also a precursor for glycine, known to relax smooth muscles, including the urinary bladder(Hong, Son, Kim, Oh, & Choi, 2005). We can extrapolate that threonine may play a role in the pathophysiology of LUTS by affecting the contractile properties of the urinary bladder. Threonine also helps maintain the health of the mucous membranes in the urinary tract by promoting mucus production, which helps protect the tissues from damage caused by acidic urine and other harmful substances(Tang et al., 2021). In addition, threonine has been shown to have anti-inflammatory properties, which can help reduce inflammation in the urinary tract caused by infections or other conditions(Manosalva et al., 2021). Threonine also plays a role in glucose metabolism and insulin signaling. Some evidence suggests that a low threonine level may be associated with insulin resistance and other features of metabolic syndrome. However, the evidence is not consistent(Guo, 2014). Threonine has been found to inhibit fat mass and improve lipid metabolism in already obese mice(Ma et al., 2020). On the other hand, a study in obese and overweight adults found that threonine supplementation did not improve insulin sensitivity, glucose metabolism, or other markers of metabolic health(Rigamonti et al., 2020).

Validating biomarkers may present challenges, particularly in measuring subtle differences in metabolite concentrations between target and control groups, the absence of targeted metabolomic experiments for follow-up, and the influence of inter-individual variation due to genetic and environmental factors (Johnson et al., 2016). To address confounding factors and identify metabolites correlated with biological processes, it is crucial to establish an appropriate experimental design and statistical power, employ questionnaires with population stratification, and utilize suitable statistical tools (Ellis et al., 2012). The analysis of metabolomics data poses significant challenges, primarily due to high data dimensionality (with numerous variables and limited samples) and the risk of overfitting the model (where the selected statistical approach overfits the training data but performs poorly on subsequent samples) (Gromski et al., 2015). Partial least squares regression is a commonly employed method for multivariate discrimination between sample classes in metabolomics analysis (Szymańska, Saccenti, Smilde, & Westerhuis, 2012).

In 2018, Mitsui et al. published the only metabolomics study to investigate male LUTS (Mitsui et al., 2018). Using the Mann-Whitney U test and multivariate logistic regression as statistical tools, they identified nine metabolites that exhibited differences between the control and LUTS groups. Specifically, they found that increased levels of glutamate and decreased levels of arginine, asparagine, citrulline, and glutamine were associated with LUTS in males. The authors postulated that abnormal glucose metabolism can be triggered as a response to starvation leading to a deceleration of the citric acid cycle. Enhanced amino acid metabolism may occur in patients with LUTS due to accelerated gluconeogenesis, supplying substances to the citric acid cycle. The urea cycle, correlated with the citric acid cycle, may also slow down in synchronization. Furthermore, reductions in arginine levels could potentially impact nitric oxide synthase. Therefore, alterations in plasma amino acid profiles may be associated with the onset of LUTS. However, it is important to acknowledge the limitations of this study, as acknowledged by the authors themselves. These limitations include the small sample size, lack of rigorous inclusion/exclusion criteria (as some patients were already on LUTS medication, which could impact the metabolomics results), and the control group primarily comprising urological patients, potentially not representing the average male in the target age group. Additionally, the study lacked appropriate statistical methods for biomarker validation, as discussed earlier.

Our study possesses several strengths. Firstly, it employed a metabolomics-based approach, enabling a comprehensive and unbiased assessment of the metabolic profile associated with lower urinary tract symptoms (LUTS). Additionally, the study implemented strict inclusion and exclusion criteria, ensuring participants were selected from the average male within the specified age group. Moreover, appropriate statistical methods, such as multiple linear regression and partial least squares (PLS), were employed to identify potential biomarkers for LUTS.

However, the study is not without limitations. Firstly, the sample size was relatively small, which may restrict the generalizability of the findings. Additionally, the study acknowledges the inherent limitations of metabolite sample preparation, which may introduce fluctuations in serum metabolite levels that are not necessarily representative of their actual variations.

In conclusion, our study highlights the importance of validation work and using appropriate statistical methods when exploring new biomarkers in urology. While Threonine emerged as a potential biomarker for LUTS through multiple linear regression, it was not corroborated by the partial least square regression. Considering the limitations of our own study and previously published research, specific patterns suggest a potential link between LUTS and disturbances in amino acid metabolism. Specifically, disruptions in gluconeogenesis from amino acids appear to be implicated. However, these findings remain inconclusive and necessitate validation in future studies with larger sample sizes and targeted metabolomics approaches. Suppose a consistent serum amino acid profile associated with LUTS can be identified. In that case, it holds promise for the development of therapeutic interventions.

.

## Data Availability

All the raw data, both the database and the metabolomics raw data is available upon request.

## References

[CR1] Cornu, J. N., Hashim, M. G. H., Herrmann, T. R. W., Malde, S., Netsch, C., Rieken, M., Sakalis, V., Tutolo, M., Baboudjian, M., Bhatt, N., Creta, M., Karavitakis, M., & Moris, L. (2023). EAU Guideline on Non-Neurogenic Male Lower Urinary Tract Symptoms (LUTS), incl. Benign Prostatic Obstruction (BPO).

[CR2] Dona AC, Jiménez B, Schäfer H, Humpfer E, Spraul M, Lewis MR, Nicholson JK (2014). Precision high-throughput proton NMR spectroscopy of human urine, serum, and plasma for large-scale metabolic phenotyping. Analytical Chemistry.

[CR3] Ellis JK, Athersuch TJ, Thomas LD, Teichert F, Pérez-Trujillo M, Svendsen C, Keun HC (2012). Metabolic profiling detects early effects of environmental and lifestyle exposure to cadmium in a human population. Bmc Medicine.

[CR4] Feinstein, L., M. B (2018). *Urologic Diseases in America. US Department of Health and Human Services, Public Health Service, National Institutes of Health, National Institute of Diabetes and Digestive and kidney Diseases*. US Government Printing Office.

[CR5] GBD. (2022). The global, regional, and national burden of benign prostatic hyperplasia in 204 countries and territories from 2000 to 2019: a systematic analysis for the Global Burden of Disease Study 2019. *Lancet Healthy Longev*, 3(11), e754–e776. 10.1016/s2666-7568(22)00213-6. 10.1016/S2666-7568(22)00213-6 PMC9640930 36273485

[CR6] Greco F, Inferrera A, La Rocca R, Navarra M, Casciaro M, Grosso G, Mirone V (2019). The potential role of MicroRNAs as biomarkers in Benign Prostatic Hyperplasia: A systematic review and Meta-analysis. Eur Urol Focus.

[CR7] Gromski PS, Muhamadali H, Ellis DI, Xu Y, Correa E, Turner ML, Goodacre R (2015). A tutorial review: Metabolomics and partial least squares-discriminant analysis–a marriage of convenience or a shotgun wedding. Analytica Chimica Acta.

[CR8] Guo S (2014). Insulin signaling, resistance, and the metabolic syndrome: Insights from mouse models into disease mechanisms. Journal of Endocrinology.

[CR9] Hong SK, Son H, Kim SW, Oh SJ, Choi H (2005). Effect of glycine on recovery of bladder smooth muscle contractility after acute urinary retention in rats. Bju International.

[CR10] Hopland-Nechita FV, Andersen JR, Beisland C (2022). IPSS bother question score predicts health-related quality of life better than total IPSS score. World Journal of Urology.

[CR11] Jiménez B, Holmes E, Heude C, Tolson RF, Harvey N, Lodge SL, Nicholson JK (2018). Quantitative lipoprotein subclass and low Molecular Weight Metabolite Analysis in Human serum and plasma by (1)H NMR spectroscopy in a Multilaboratory Trial. Analytical Chemistry.

[CR12] Johnson CH, Ivanisevic J, Siuzdak G (2016). Metabolomics: Beyond biomarkers and towards mechanisms. Nature Reviews Molecular Cell Biology.

[CR13] Koeth RA, Wang Z, Levison BS, Buffa JA, Org E, Sheehy BT, Hazen SL (2013). Intestinal microbiota metabolism of L-carnitine, a nutrient in red meat, promotes atherosclerosis. Nature Medicine.

[CR14] Kozminski MA, Wei JT, Nelson J, Kent DM (2015). Baseline characteristics predict risk of progression and response to combined medical therapy for benign prostatic hyperplasia (BPH). Bju International.

[CR15] Lee SWH, Chan EMC, Lai YK (2017). The global burden of lower urinary tract symptoms suggestive of benign prostatic hyperplasia: A systematic review and meta-analysis. Scientific Reports.

[CR16] Ma Q, Zhou X, Sun Y, Hu L, Zhu J, Shao C, Shan A (2020). Threonine, but not lysine and methionine, reduces Fat Accumulation by regulating lipid metabolism in obese mice. Journal of Agriculture and Food Chemistry.

[CR17] Manosalva C, Quiroga J, Hidalgo AI, Alarcón P, Anseoleaga N, Hidalgo MA, Burgos RA (2021). Role of Lactate in inflammatory processes: Friend or foe. Frontiers in Immunology.

[CR18] Mitsui T, Kira S, Ihara T, Sawada N, Nakagomi H, Miyamoto T, Takeda M (2018). Metabolomics Approach to male lower urinary tract symptoms: Identification of possible biomarkers and potential targets for New Treatments. Journal of Urology.

[CR19] Mullins C, Lucia MS, Hayward SW, Lee JY, Levitt JM, Lin VK, Getzenberg RH (2008). A comprehensive approach toward novel serum biomarkers for benign prostatic hyperplasia: The MPSA Consortium. Journal of Urology.

[CR20] Newgard CB (2017). Metabolomics and metabolic Diseases: Where do we stand?. Cell Metab.

[CR21] Oliver SG, Winson MK, Kell DB, Baganz F (1998). Systematic functional analysis of the yeast genome. Trends Biotechnol.

[CR22] Patel DN, Feng T, Simon RM, Howard LE, Vidal AC, Moreira DM, Freedland SJ (2018). PSA predicts development of incident lower urinary tract symptoms: Results from the REDUCE study. Prostate Cancer and Prostatic Diseases.

[CR25] Rajalahti T, Kvalheim OM (2011). Multivariate data analysis in pharmaceutics: A tutorial review. International Journal of Pharmaceutics.

[CR23] Rajalahti T, Arneberg R, Kroksveen AC, Berle M, Myhr KM, Kvalheim OM (2009). Discriminating variable test and selectivity ratio plot: Quantitative tools for interpretation and variable (biomarker) selection in complex spectral or chromatographic profiles. Analytical Chemistry.

[CR24] Rajalahti T, Kroksveen AC, Arneberg R, Berven FS, Vedeler CA, Myhr KM, Kvalheim OM (2010). A multivariate approach to reveal biomarker signatures for disease classification: Application to mass spectral profiles of cerebrospinal fluid from patients with multiple sclerosis. Journal of Proteome Research.

[CR26] Rigamonti, A. E., Leoncini, R., De Col, A., Tamini, S., Cicolini, S., Abbruzzese, L., & Sartorio, A. (2020). The appetite-suppressant and GLP-1-Stimulating Effects of Whey Proteins in obese subjects are Associated with increased circulating levels of specific amino acids. *Nutrients*, *12*(3), 10.3390/nu12030775. 10.3390/nu12030775 PMC7146343 32183423

[CR27] Roehrborn CG (2008). BPH progression: Concept and key learning from MTOPS, ALTESS, COMBAT, and ALF-ONE. Bju International.

[CR28] Speakman M, Kirby R, Doyle S, Ioannou C (2015). Burden of male lower urinary tract symptoms (LUTS) suggestive of benign prostatic hyperplasia (BPH) - focus on the UK. Bju International.

[CR29] Szymańska E, Saccenti E, Smilde AK, Westerhuis JA (2012). Double-check: Validation of diagnostic statistics for PLS-DA models in metabolomics studies. Metabolomics.

[CR30] Tang, Q., Tan, P., Ma, N., & Ma, X. (2021). Physiological functions of threonine in animals: Beyond Nutrition Metabolism. *Nutrients*, *13*(8), 10.3390/nu13082592. 10.3390/nu13082592 PMC8399342 34444752

[CR31] Wang W, Guo Y, Zhang D, Tian Y, Zhang X (2015). The prevalence of benign prostatic hyperplasia in mainland China: Evidence from epidemiological surveys. Scientific Reports.

